# Correction: Gender imbalances in the editorial activities of a selective journal run by academic editors

**DOI:** 10.1371/journal.pone.0297480

**Published:** 2024-01-17

**Authors:** Tal Seidel Malkinson, Devin B. Terhune, Mathew Kollamkulam, Maria J. Guerreiro, Dani S. Bassett, Tamar R. Makin

The first author, Tal Seidel Malkinson, is incorrectly noted as the corresponding author. The correct corresponding author is Tamar R. Makin. Tamar R. Makin’s email address is: tamarmakin@gmail.com.
