# Supplementary figures and images for: Gender imbalances in the editorial activities of a selective journal run by academic editors
Source: PLoS One. 2023 Dec 11;18(12):e0294805. doi: 10.1371/journal.pone.0294805 (PMC10712860; doi:10.1371/journal.pone.0294805)

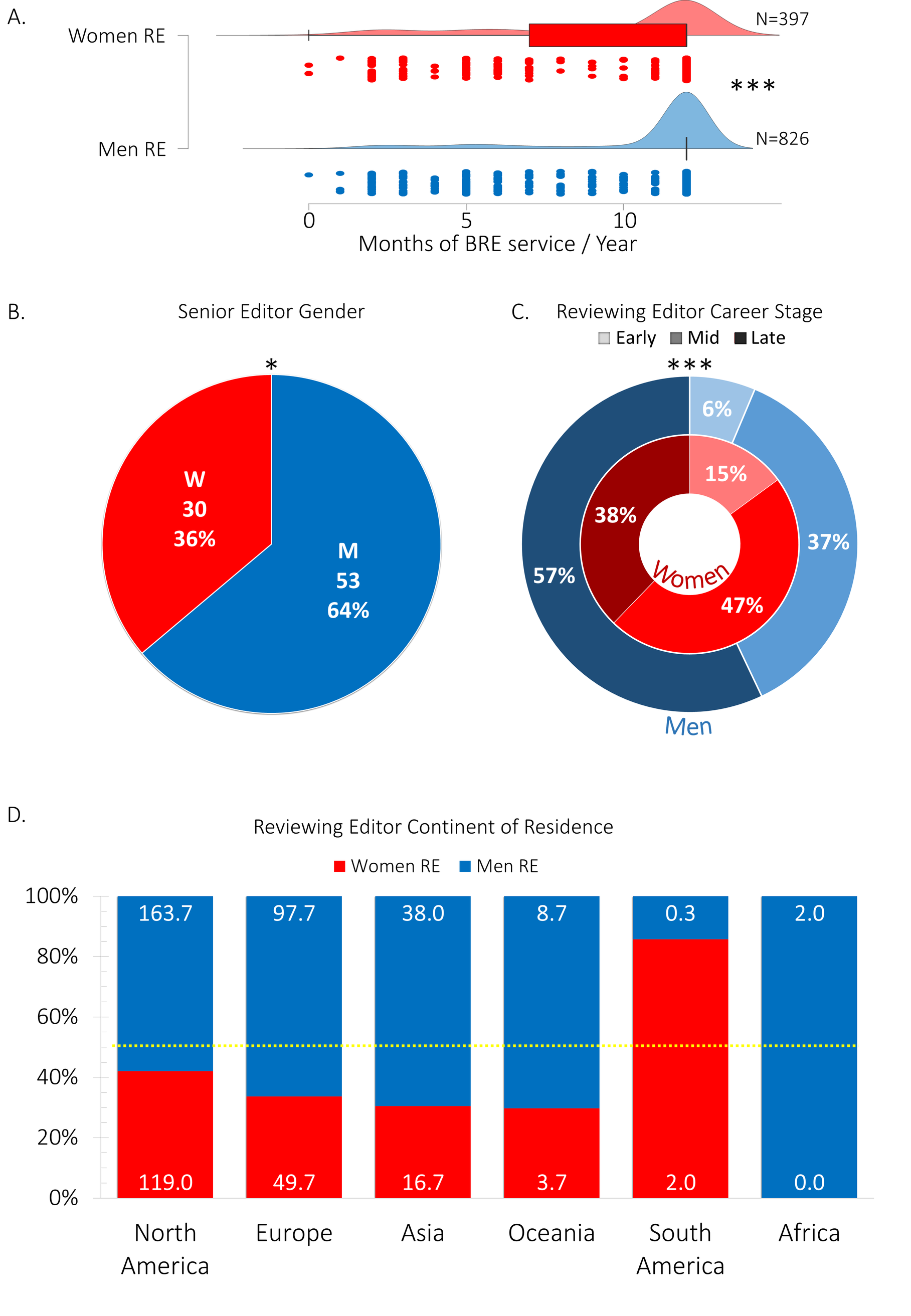

Supplement: S1 Fig — A. Women Reviewing Editors (N = 397) serve on average slightly fewer months per year as active BRE members than men (N = 826) do, throughout 2017–2019. B. Senior Editor gender base rate. In 2021 there were significantly more men (N = 53) than women (N = 30) Senior Editors, as indicated by the asterisk. C. Men and women Reviewing Editors career stage. Compared to men REs, women REs were at earlier career stages, as indicated by asterisks. Note that these findings are based on data that was sampled at a different time point than our main datasets, and thus cannot be directly linked to the main findings. D. Reviewing Editor continent of residence. Numbers indicate the mean number of women and men REs from each continent across the three datasets (February 2019, January 2020 and December 2020); dashed yellow line depicts gender balance (50%). There was no evidence for gender disparity in the geographical representation of women and men REs. A-C. Men-blue, women-red; *p≤0.05, **p≤0.01, ***p≤0.001. (TIF) [file pone.0294805.s001.tif]

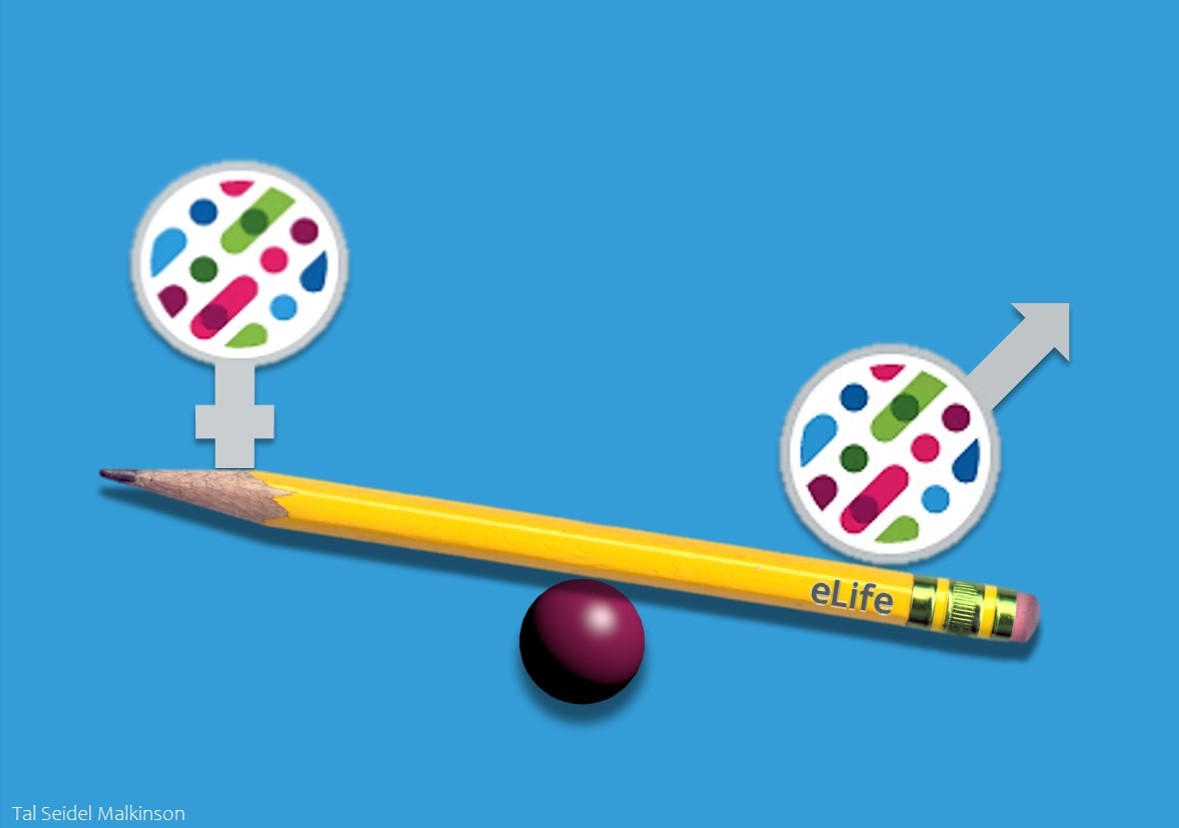

Supplement: S2 Fig — (JPG) [file pone.0294805.s002.jpg]
