# Supplementary material for: Gender imbalances in the editorial activities of a selective journal run by academic editors
Source: PLoS One. 2023 Dec 11;18(12):e0294805. doi: 10.1371/journal.pone.0294805 (PMC10712860; doi:10.1371/journal.pone.0294805)
Supplement: S1 File — (DOCX) [file pone.0294805.s003.docx]

# Supplementary data

## Supplementary methods

### Datasets

To get some intuition about potential demographic factors that could mitigate gender disparities observed in our data, we analysed two additional datasets.

### Senior Editor Dataset – This dataset contains information relating to the assigned gender (as described in the Methods section) of eLife Senior Editors, including Editor-in chief and Deputy Editors, who act as Senior Editors in the reviewing process. These data were extracted from eLife’s website (eLife leadership team (2021), retrieved from <https://elifesciences.org/about/people>).

BRE demographic dataset – This dataset contains anonymous information relating to the assigned gender of REs (as described above), their continent of residence, as inferred by the location of the institution where they are primarily based, and their career stage (number of years since independence). These analytic data were acquired by eLife during February 2019, January 2020, and December 2020. RE career-stage was divided into three categories: Early career (less than or equal to 5 years of independence), Mid-career (6-15 years of independence), and Late career (more than 16 years of independence).

### Statistical analysis

*N*-1 χ^2^ proportion comparison test was performed to compare the gender proportions of Senior Editors MedCalc online tools (MedCalc Software, Ostend, Belgium), and contingency table analysis was used for testing the interrelation between RE gender and Career stage, and between RE gender and Continent of residence, using JASP software.

## Supplementary results

Woman REs served on average less months per year as active BRE members compared to men REs (Women: 9.65±3.60 months of service per year; Men: 10.52±2.97 months of service per year; Welch t-test: t(663.91)=4.18, *p*<0.001, Hedges’ *g*=0.26; Supp Fig 1A). This difference might reflect eLife’s progressive efforts to increase the number of women REs over the months of the year.

There was a significant imbalance in Senior Editor’s gender (36% women vs. 64% men, *χ*2_(1)_=5.848, *p*=0.016, Cohen’s *h*=0.56; Supp Fig 1B).

There was a significant disparity in the career stage distribution between men and women REs: women REs tended to be at earlier career stages than men REs (women: Early 14.95%, Mid 47.27%, Late 37.78%; Men: Early 6.34%, Mid 36.60%, Late 57.06%; *χ*^2^_(2)_=56.04, *p*<0.001, Contingency coefficient=0.20; Supp Fig 1C). In contrast, there was no evidence for gender disparity in the geographical representation of women and men REs (*χ*^2^_(5)_=8.36, *p*=0.14; Supp Fig 1D).

Note that these findings are based on data that was sampled at a different time point than our main datasets, and thus cannot be directly linked to the main findings.
