# Supplementary material for: Gender imbalances in the editorial activities of a selective journal run by academic editors
Source: PLoS One. 2023 Dec 11;18(12):e0294805. doi: 10.1371/journal.pone.0294805 (PMC10712860; doi:10.1371/journal.pone.0294805)
Supplement: Attachment — Submitted filename: Response to Reviewers.docx [file pone.0294805.s004.docx]

Response to Reviewers

Reviewer #1: The study aims to determine whether the involvement of individuals in elife’s board of review editors is subject to gender disparities at various stages of the editorial process. The study discovered gender imbalances in the various stages of the editorial process of eLife. This study is interesting, and the analysis is sound.

**Major concern:**

(1) It would be beneficial to state clearly why editorial month was chosen over individual editor count to calculate the gender composition of REs.

Thanks you for pointing at this confusing issue. Since we were dealing with a dataset acquired over 3 years, we wanted to reflect fluctuations in the involvement of editors over this period. For example, some reviewing editors were enlisted close to the end of a given year, meaning they did not have the same opportunity to contribute to the editorial activities. Since during the studied period the representation of women in the BRE increased, it was particularly important to reflect partial contributions correctly. We chose months of BRE service as a practical unit that reflects partial work time:

*To account for variability in BRE service contract durations, we used for this purpose the number of months in which the RE was affiliated with the BRE per year (months of BRE service; see methods section for further details).*

*In order to measure BRE members service contribution as a function of gender, we computed the proportion of months of service of women and men REs out of the total number of service months, thus taking into account partial work time (e.g. REs appointed in the middle of the year, being on leave).*

In the revised manuscript, to avoid further confusion we changed our terminology from ‘months of BRE service’ to ‘service’, and provide full details of this measure in the methods.

(2) Page 10 regarding the search of keywords in PubMed: How was the search done? I imagine there will be a lot of keyword overlap among REs. Welch’s t-test works for independent samples, while the search results are not necessarily independent?

In the keyword analysis, we counted the number of PubMed publications that could be associated to the proclaimed expertise of each RE. For example, the list of keywords provided by a specific RE (age-related macular degeneration, diabetic eye disease, retinopathy, ocular disease) was strung together using the OR operator (“age-related macular degeneration OR diabetic eye disease OR retinopathy OR ocular disease”) and queried against PubMed using the API, returning 215,250 search results (hits). This was a mean to estimate the scope and reach of the specific range of expertise, as provided by each of the REs. It is true that there is substantial overlap across REs, yet each individual was invited to provide their own set of keywords, meaning no two REs had the identical combination of terms. It might be worth highlighting that there was no initial database of terms, or suggested terms provided by eLife, meaning each RE was asked to come up with their own string of terms.

Because keywords for expertise were self-defined, the specific terminology used by men and women could have been relevant for our research. Indeed, there is other research to show that men tend to use broader terms then women when discussing their expertise (Kolev et al., 2019). For example, given expertise in visual processing, the term ‘perception’ will have a broader scope than the word ‘vision’. We therefore felt obliged to determine whether this factor could have contributed to authors’ tendency to suggest more man than women as potential BREs at the Initial Submission stage.

The number that we fed into the Welch test was the unique number of PubMed publications that each RE query yielded. As such, these units were independent from one another, even if the same term (e.g. vision) was used as part of multiple strings. For this reason, we are not concerned about violation of the assumption of the statistical test.

In the revised submission, we have taken a few steps to improve how this information is being communicated.

(1) We provided some further details of the operation in the results section:

Specifically, previous research points at potential disparity with the broadness of term women and men use when communicating research (Kolev et al., 2019). *Accordingly, we quantified the number of PubMed search results for women and men BRE members’ keywords. Specifically, we queried the number of PubMed publications associated with the string of keywords provided by each RE, using an ‘OR’ operator. This provided us with a simple mean of scope.*

(2) We added missing information in the methods, when describing the dataset, about the fact that the keywords were self-generated:

*In addition, the editorial staff asks REs to provide a set of keywords that reflect the scope of their research, which was also included in this dataset. Note that the terminology was self-generated by the Res (rather than adopted from an existing database), and that there were no limitations on the number of keywords each RE could provide.*

(3) We separated the keyword analysis using a separate sub header in the methods to make it easier for the reader to obtain the relevant information.

(3) Regarding the gender difference in full submissions: I think it would be important to see if there is any gender difference in the percentage of initial submissions invited for full submissions handled by REs. This could be related to the observed differences in the number of full submissions handled by women and men REs.

We agree with the reviewer that it would have been highly informative to learn if the decision to send a manuscript for review could have been affected by the gender of the REs assessing the Initial Submission. However, this is not a simple question to quantify based on our available database. First, we were working with two different datasets, one of which was based on individual manuscripts, the other on the REs. These were not linked with each other, meaning we did not have information about the REs involved in the Initial Assessment of each manuscript. In other words, we do not have the gender composition of the teams working during Initial Assessment. Beyond this technical limitation, it is important to emphasize that the initial decision is made by the SE based on a consultation with teams of REs, which are invited to contribute to the discussion. In this context, not all team members will contribute equally (this is something that we raised in the analysis pertaining to women’s availability to respond to the initial consultation requests and its trickle-down effects). For this reason, it becomes extremely complicated to assign gender contributions to group dynamics, and a further motivation for us to focus on the gender dynamics when only two editors are involved (SE and RE) during the Full Submission stage. This is a limitation of investigating a real-life process, we are simply unable to address this interesting and important question.

(4) I understand that the study aims to capture the gender differences among the editorial activities of eLife. But it is hard to provide helpful insights to the related communities without knowing how the situation of eLife looks like compared with other journals, and how this relates to the outcomes of eLife’s editorial process – the publications. Does the presented gender imbalance in the editorial process make a difference in the overall scholarly communication system by favoring/disfavoring a particular group of authors?

Multiple studies have shown that across different scientific fields, women’s representation in editorial boards of scientific journals is smaller than men’s representation, e.g. 40% in journals in the field of Psychology and 30% in Neuroscience (Palser et al., 2022), 9% in the field of Mathematics, and 26% across the Frontiers publishing group (Helmer et al., 2017). Our findings of under-representation of women REs in eLife’s BRE (31% women REs and 36% women SEs) is in line with these findings. Hence, our unique access to gender dynamics in the internal editorial decision process of eLife’s BRE should also be pertinent to other scientific journals, who suffer from similar gender disparities among their editorial teams.

Regarding the outcomes of the biases we identified, this is something that we are happy to speculate about, with relation to highly related publications. For example, a study involving 9,000 editors and 43,000 reviewers across the Frontiers publishing group have also shown homophily effects when assigning reviewers (Helmer et al., 2017). Moreover, a previous study of eLife editorial decisions focused on how the gender makeup of the participants in the peer-review stage – both editors and reviewers ­­– biases acceptance rates for men and women authors, found that all-men reviewer teams are far more likely to accept men-led manuscripts (Murray et al., 2019). Therefore, the homophilic behaviour that we observe at the stage of appointing REs, is likely to exacerbate homophily effects and increase the gender publishing gap. Crucially, as we explain the manuscript, the homophily effects work in the favour of men, due to historical and trickle-down disparities, which have been tracked all the way up to the makeup of editorial boards (Palser et al., 2022).

In the revised manuscript we added the following paragraph to our conclusions section:

*We find multiple consistent disparities across the editorial process, which culminate in the disproportional handling of submissions for peer review by men relative to women, even after taking into consideration men’s over-representation in eLife’s BRE. This effect was not a mere consequence of different gender distribution across disciplines, meaning it is not due to lack of available expert women, but rather a tendency of men SEs to favor men REs over women REs. This homophily effect is known to influence editorial decision-making, e.g. in recruitment of reviewers (Helmer et al., 2017) and in favorable evaluation of manuscript led by men (Murray et al., 2019). Therefore, it is easy to speculate that the disparity effects we observed here would be further amplified as the decision process trickles down. In other words, the gender disparity that tends to disfavor women cannot be pinpointed to a single stage in the editorial evaluation process, but should instead be viewed as a systematic accumulation of biases across multiple decision-making steps of a people-led process.*

**Minor:**
(1) Captions for tables vary across the manuscript.

Corrected with thanks.

(2) Page 13 line 259 referred to Figure 2E, which was not available in the manuscript

Corrected to Figure 1E, with thanks.

(3) Page 32 Table 1: The bottom table seems incomplete?

We apologize for the confusion, Table 1 bottom table is complete and shows the composition of eLife’s Manuscript dataset, which included two fields of chosen discipline terms for each manuscript, such that manuscript authors chose up to 2 descriptive discipline terms, out of a list of 18 possible terms, to describe their manuscript. This was clarified in the revised Table legend:

*Manuscript Dataset: … It includes the following fields: … The two discipline terms the authors chose, out of 18 possible terms (Discipline 1 & Discipline 2; see Table 2 for details).*

## Reference

Kolev, J., Fuentes-Medel, Y., & Murray, F. (2019). *Is Blinded Review Enough? How Gendered Outcomes Arise Even Under Anonymous Evaluation* (No. 25759). National Bureau of Economic Research. https://doi.org/10.3386/w25759

Reviewer #2:

**Major Comment for Authors**

Yours is an excellent introduction on the impact of publication in the lives of academics and consequences of bias. However, you omitted an important issue—the topic of the manuscript may influence its publication in a gender-biased way. In addition, you have the opportunity to assess this given the open and huge database eLife has made available (to their credit). I feel that the current tone of the manuscript is justification of the status quo. It may be that removing of paid eLife employees will decrease that perception. This is an important and serious criticism that must be rectified.

We thank the reviewer for her positive assessment. In the revised manuscript, we speculate on how our present findings link with other known homophily effects to create systematic gender biases that favour men. We paint this status quo, not to condone but to rectify. Due to the style of a research article, we are committed to adhering to a scientific style that is based on our own findings (and their relations to other published findings), and reserve our opinions to a more adequate format. Indeed, we have found that this research has evoked a range of reactions, and in fact this study was previously rejected from publication in another journal (PLoS Biology) by a reviewer who suggested that we are blaming men for women’s inadequacies:

*“The authors don’t directly make this link, but just* ***bemoan*** *the fact that men handle more cases…If so, why were women “under-engaged” i.e. handling fewer articles? Well, the authors show (lines 164/170) that women RE’s were less likely to respond to the SEs’ initial consultation request and took longer to respond. … If so, we cannot* ***blame*** *the Ses for the fact that “women editors … were under-engaged in editorial activities”, and we cannot refer to this as bias.”*

Our solution to this tension is constructive feedback. Throughout the manuscript we constantly provide suggestions and recommendations for how the biases identified in our study could be rectified (this is summarized in Table 3). We hope that the additional paragraph we added to the conclusions section helps clarify our position:

*We find multiple consistent disparities across the editorial process, which culminate in the disproportional handling of submissions for peer review by men relative to women, even after taking into consideration men’s over-representation in eLife’s BRE. This effect was not a mere consequence of different gender distribution across disciplines, meaning it is not due to lack of available expert women, but rather a tendency of men SEs to favor men REs over women REs. This homophily effect is known to influence editorial decision-making, e.g. in recruitment of reviewers (Helmer et al., 2017) and in favorable evaluation of manuscript led by men (Murray et al., 2019). Therefore, it is easy to speculate that the disparity effects we observed here would be further amplified as the decision process trickles down. In other words, the gender disparity that tends to disfavor women cannot be pinpointed to a single stage in the editorial evaluation process, but should instead be viewed as a systematic accumulation of biases across multiple decision-making steps of a people-led process.*

Specific necessary changes:

1. Justify inclusion of paid eLife employees in this publication’s authorship.

The only paid eLife employee is Maria Guerreiro, who was the Head of Journal Development at eLife. It is due to her commitment, time and hard work that we were able to conduct the study. Maria curated the data for us, a complicated process that required her to consider our unique requirements (e.g. GDPR ethics requirements). By any standard, this qualify Maria as a contributing author, and her conflict of interest was clearly stated as part of the submission process. It might reassure the Reviewer to learn that this ‘side’ project was not a part of Maria’s formal responsibilities with eLife, and that she has recently moved to a new organisation (EMBO).

The senior author of the manuscript (Professor Tamar Makin) is a Senior Editor with eLife, a role that is not a part of eLife’s executive team. Her involvement in the study did not relate to her paid responsibilities with eLife (which is handling submissions), but rather her role as a researcher with UCL and the University of Cambridge. Her in-depth understanding of eLife’s editorial process was essential for coming up with the research plan, analysis and write up of the manuscript, which she initiated. These roles qualify her as senior author, and her conflict of interest is clearly reported.

It is important for us to highlight that while eLife has given us access to the data, they did not fund our research, nor did they provide us with any additional requirements. In fact, they have been helpful and facilitating of our aim to research gender biases in their editorial process. Nevertheless, to avoid the appearance of a conflict of interest, we decided to publish our manuscript in an outlet not related to eLife (PLoS One).

2. Add to this analysis a review the issue of topic as a potential source of gender-related bias in this journal’s publication record. That has only been shown in biomedical publication in general medical journals but may also be true in other aspects of science.

This is an excellent suggestion, however – unfortunately – due to GDPR constraints eLife were required to anonymize all data prior to sharing it with us. This means we were not privy to the topics (e.g. titles) of the manuscripts we were assessing. We did, however, receive information relating to the scientific field of each submission in our manuscript database. This allowed us to examine whether the main study finding – the homophily effect – was related to the proportion of manuscript assignment to women in each field, thus allowing us to assess this point on a coarser scale. The results of this analysis are reported in Figure 2. In short, we found a significant homophily effect of Senior Editor gender on the gender of the assigned RE in 14 out of the 18 disciplines (see Table 2 for details). These results demonstrate that gender homophily in manuscript assignment is a widespread cross-disciplinary effect, naturally spanning many different topics. We read your manuscript with interest, and have now included it in our Introduction and Discussion.

Reference List

1. Kalidasan D, Goshtasebi A, Chrisler J, Brown HL, Prior JC. Prospective analyses of sex/gender related publication decisions in general medical journals: editorial rejection of population- based women’s reproductive physiology. BMJ Open 2022. DOI: 10.1136/bmjopen-2021-057854.
