# Supplementary material for: Gender imbalances in the editorial activities of a selective journal run by academic editors
Source: PLoS One. 2023 Dec 11;18(12):e0294805. doi: 10.1371/journal.pone.0294805 (PMC10712860; doi:10.1371/journal.pone.0294805)
Supplement: Attachment — Submitted filename: Plos one Rev 2 rebuttal letter (1).docx [file pone.0294805.s005.docx]

**Reviewer #2: Major Comment for Authors**

I understand the roles of the authors who work with eLife much better—what you put in explanation to me needs to be in the manuscript, however. I also do now see that you are trying to improve equity. But it still feels to me like there is bias in the reporting/writing and the interpretation of these results. Likewise, the conclusions in Table 3 are too non-specific and vague to be of any real assistance in achievement of equity.

**Reply**

We thank the reviewer for her sincere efforts to help us improve the manuscript. However, we respectfully disagree with the Reviewer’s statement that we introduced biases in the reporting, writing and the interpretation of the results. We have made every effort to faithfully interpret our findings in a transparent and balanced way, and believe our interpretation reflects the data which we carefully analyzed based on best statistical practices. Below we elaborate on how decision process, in lieu of the reviewer’s comments.

Regarding Table 3, we acknowledge that the recommendations may lack specificity and might not fully attain equity. Our intention was to provide valuable guidance for stakeholders seeking a proactive approach in pursuing that objective. In the revised manuscript, we have taken greater care to provide a clearer explanation of the purpose of Table 3, which is outlined as follows:

In the Conclusion:

*Table 3 provides a summary of our results and aims to offer potential guidance to stakeholders for taking a proactive approach towards enhancing gender equity in editorial activities.*

In Table 3 legend:

*The proposed solutions aim to provide potential guidance to stakeholders, enabling them to adopt a proactive approach towards enhancing gender equity in editorial activities.*

**Specific Necessary Changes**

1. Please add the explanations (like you made in response to my critique) of the roles of the authors that are officially affiliated with eLife.

**Reply**

We have now included a more comprehensive conflict of interest statement, as follows:

*Maria Guerreiro was the Head of Journal Development at eLife and was the sole paid eLife employee involved in this study. Her contribution was essential for conducting the research, including data curation in line with the study's specific requirements. The senior author, Professor Tamar Makin, serves as a Senior Editor at eLife. Her involvement in this study was independent of her paid responsibilities at eLife. Leveraging her understanding of eLife's editorial process, she played a vital role in developing the research plan, conducting analysis, and writing the manuscript*

2. In all statistical analysis, use judgement in interpreting the data based on the numbers in the analysis and an a priori understanding of what is important. For example, the paper is littered with references to “less responsive” women. That is based, in part, on this statistic: A response of 88% by women versus by men of 91% does not seem important to me (line 201). When small differences are statistically significant in a large data, that conclusion must be qualified.

**Reply**

We acknowledge and appreciate the reviewer's feedback, and as a result, we have revised our language to ensure a more neutral tone. We replaced the wording “reduced responsiveness” with “longer response time and less frequent responses”, which describes the findings more closely, and is not an attribution to the women REs.

Regarding the statistics, we assure the reviewer that careful consideration and expertise were applied in making these decisions. Several authors possess relevant expertise as statistics lecturers and advocates of best statistical practices. We acknowledge, both in this statement and in the text, that the observed effects may be subtle, as exemplified by the reviewer's provided example. However, it is worth noting that even these modest effects can have cascading consequences. For instance, a slight delay in response could potentially influence the subsequent tone set by another reviewing editor during a consultation. This is explained here:

*Women took only slightly longer to respond relative to men* *(women were approximately 7 hours slower to answer emailed invitations), but considering the interactive nature of the consultation process, this delay could be meaningful. In the eLife initial consultation process, where editors’ interact in an on-line instant chatting format, this means that men are more likely to set the tone of the discussion by providing their opinion first, making it more difficult for women, on average, to influence the editorial decision (through conformity and anchoring cognitive biases for example [86–88]). It has been previously shown that it is more difficult to voice a different opinion once an opinion has been formed [89,90].*

3. The “unresponsive women” narrative is also based on this statistically significant difference in the time to respond to the request: 1.83±1.55 days vs. 1.54±1.23 days (line 203). Would a rational person consider three tenth of a day an important delay?

**Reply**

The unfortunate truth is that a small but significant disparity in response time during a consultation carries tangible consequences downstream. Specifically, in the eLife initial consultation process, where editors’ interact in an on-line instant chatting format, this small delay means that men are more likely to set the tone of the discussion by providing their opinion first, making it more difficult for women, on average, to influence the editorial decision. This example is of utmost significance as it highlights a systemic bias that disproportionately affects women, who, as we have previously emphasized, often face greater commitments compared to men. This realization motivated us to propose concrete suggestions in Table 3: in order to provide women with an equitable opportunity to influence the editorial decision process, it is essential to take into account the delayed responses we have observed for women.

4. The observation that women editors are earlier in their careers is key and should be used in interpretation of all of the data. Also, it essential, to compare the time and contributions of the senior women academics with senior men academics. That doesn’t appear to have been done and must be.

**Reply**

We sincerely regret that we will not be able to accommodate this suggestion. As explained, the data we collected to demonstrate career stage was taken at a different time point and we are unable to make direct links between the effects we report in the main study. We emphasized the significance of this crucial consideration and this limitation in the Discussion in the Datasets section and the :

*However, the specific factors that we could study were not pre-determined based on our experimental needs. Accordingly, we were limited in our explanatory power, both in terms of other relevant factors that might be contributing to the observed effects (e.g., the level of seniority of each RE) and in terms of the statistical power (e.g., authors’ appeals are rather infrequent). To mitigate some of these gaps, we can gain some insight from more recent data relating to the heterogeneity of eLife’s BRE (see Supplemental Section, Fig. S1), although these recent analytics may not fully represent the dataset we analysed here. It is also important to consider the makeup of the BRE; these are invited roles, and as such, all the REs are established in their subfields. However, due to issues we expand upon below, it is possible that women REs are less senior than men REs, as described in the Supplemental data. … With these points in mind, our gender effects might be modulated by other contributing factors (such as seniority, identity that should be investigated in future research in greater detail.*

We now expanded on this point in the revised Supplementary results and Supplementary Figure 1C:

*Note that these findings are based on data that was sampled at a different time point than our main datasets, and thus cannot be directly linked to the main findings.*

5. You could compare variables between the early and mid-career women and same academic-stage men, but should acknowledge that results may be biased by urgent domestic responsibilities that younger women often have.

**Reply**

We have reiterated this important consideration in the discussion (see below). We would like to emphasize that domestic responsibilities are an important potential factor, but not the only one. Research shows that women researchers who are not overburdened with domestic responsibilities (e.g. who have advanced in their careers beyond childcare age, or who are childfree) still experience biased treatment. For example, senior women scientists tend to be overburdened with administrative responsibilities compared to their male colleagues (Winslow & Davis, 2016; Wright et al., 2003), and childfree women are negatively stereotyped, experience more workplace incivility than other employees ([Verniers](https://www.researchgate.net/profile/Catherine-Verniers?_sg%5B0%5D=ASAeEtRoL96oIznz56Xe57Rj7zuScX3Fud2MarFDDkdff0sGKVZt1aQ0TGxMhzIOjdFTzDg.1cd3SFtqt5YAHiV8Et4N6WcNR2VVlRneLmmb5Gc0GADzPsDQTlM8KvaF-VK6MCN_DuhOdPeU5LPQs_T23dbqjQ&_sg%5B1%5D=Bi6svoDjJ9N3q70PuZiPzyNxzBPmGeP_QiANQnVwkmBjdbQrhxrfH2f2GMbIpJyiruEqaWE.Vdl07Gpwu-o_gQkzbPipJew8uDpCSbaxQUVwNjK-mvaTjeZP73-ZYg5R6L7370sBBSmwGyibjgURZAOHlwMdTw), 2020), and are disadvantaged in advancement in their scientific careers compared to men (Morgan et al., 2021; Santos & Dang Van Phu, 2019). Gender bias has very deep and complex roots, and we wanted the discussion to reflect the range of different causes that affect women, whoever and wherever they are. We are confident that the reviewer will support this ambition.

*Women’s longer response time, as well as less frequent response rate (by approximately 3%) could potentially be attributed to the fact that women have more duties and responsibilities than men REs. There are multiple reasons to suggest this, depending on women’s specific intersecting identities [43,91,92]. For example, senior women are overburdened by administrative responsibilities due to the institutional need to narrow the gender gap [25,30]. More specifically to our dataset, there is a hint that women REs are at an earlier career stage relative to men (Supplementary section), and hence may be more likely to have children at home than their men colleagues and thus face an added burden on their time [43], or be more laden with obtaining tenure. Another potential contributing factor is the higher standard of communication women are held to in order to receive equal acknowledgment, resulting in an imposed time-consuming quantity/quality trade-off for women, and reducing their productivity [47,93,94]. Irrespective of the reasons, our results signal that the journal submission and review process needs to shift away from monitoring decisions based on the decision time, which adds time pressure, and instead could potentially delay discussion and/or decisions about submissions until women have contributed.*

6. Another place where this paper itself feels gender biased is in the homophily responses by the different disciplines, it is true that it is present in both men and women editors. However, this tendency to choose men if you are a man editor is much greater (77%) than a women’s tendency to choose women (41%)-- lines 274-275. No comment is made that this proportion of assignment to same-sex reviewers in statistically different—but it certainly looks like it is! Please correct.

**Reply**

Thank you for bringing up this important point. However, the numbers cited by the reviewer do not account for the base rate of manuscript assignment to women and men REs. When considering the gender of SEs, we found that women SEs assigned to women REs 41.41% of the manuscripts, compared to 30.04% manuscript assignment to women when not taking into account the SE’s gender. Conversely, men SEs assigned to men REs 76.57% of the manuscripts, compared to 69.96% manuscript assignment to men when not taking into account the SE’s gender. This indicates an over-assignment effect of 11.37% for women, while men had an over-assignment effect of 6.61%. These effects have been statistically quantified in the contingency tables analysis (line 219), demonstrating a significant small-to-medium effect size. Importantly, this effect amplifies the disproportionate allocation of manuscripts to women REs over and above their under-representation in the BRE. We hope that this clarification puts the Reviewer at rest that we did not neglect to report any gender biases in our dataset.

7. I like the constructive idea that the duration of a review needs to allow time for responses from both women and men.

**Reply**

We thank the Reviewer for her positive feedback. We hope that this adds value to the analysis we discussed in points 2-3 above.
