# Supplementary material for: Gender imbalances in the editorial activities of a selective journal run by academic editors
Source: PLoS One. 2023 Dec 11;18(12):e0294805. doi: 10.1371/journal.pone.0294805 (PMC10712860; doi:10.1371/journal.pone.0294805)
Supplement: Attachment — Submitted filename: plos one rebuttal letter to reviewers 3.docx [file pone.0294805.s006.docx]

Reviewer #2: PONE-D-22-28312R2 eLife evaluation of gender related to academic publication

Major Comment for Editor

Thank you for sending me the document with the minimal track changes by the authors. I understand that I need to be more specific in what I request that they do.

The responses to my comments from the authors were largely appropriate. The paper is also reviewed with the new Table (3).

However, still the same lingering rather pompous-feeling masculine bias wafts over the manuscript.

**Reply**

We appreciate the reviewer's constructive feedback aimed at improving our manuscript. However, we would like to express our concern about the tone and some of the comments in the review, which may appear unfounded and subjective.

Our commitment to promoting equity, diversity, and inclusion is deeply ingrained in our research objectives, and this commitment served as the driving force behind our study. While we fully acknowledge the importance of addressing gender equity in research, it is equally critical to ensure that any modifications made to the manuscript are grounded in sound statistical principles and accurately represent our findings.

We want to emphasise that the conclusions drawn from our data are firmly based on statistical analysis and should not be confused with bias. Our intention is to provide a comprehensive and data-driven perspective on the subject, and we welcome further suggestions to enhance the manuscript in this regard.

There are also some English editorial changes that are needed, such as italicizing eLife and breaking up very long and detailed paragraphs.

**Reply**:

We appreciate the reviewer's suggestion. However, we believe these issues can be addressed as part of the proofs with the relevant editor.

My suggestions need to actually be made in the manuscript and limitations listed in the Discussion. It is not sufficient that they are acknowledged in the response to reviewer document. For these reasons I’m making line-specific requirements, providing you agree, for changes in the manuscript and in the new Table 3.

I want to see this paper published. It is comprehensive and important scholarship. However, the gender bias subtleties must be respected and where they have crept in, must be removed.

**Reply**:

Below we provide some additional clarifications to contextualise some of our key decisions concerning the revisions. Whenever possible, we have included further revisions.

Major Comment for Authors

Thank you for your positive responses to and acknowledgement of my suggestions. However, these need to make their way into the manuscript itself for the feeling of gender bias to be removed from this important piece of work.

Specific, necessary manuscript changes

1. Abstract—lines 8 to 9 Add a period after “submissions.”

Remove what is now there.

The next sentence should read: “However, women Reviewing Editors (RE) are less engaged related to these submissions, perhaps because they enter the online editorial discussions slightly (7 hours) later, and in subsequently obtained data have systematically lower academic ranks than the men RE.”

**Reply**:

We appreciate the reviewer's suggestion. However, it is important to note that the information about academic rank is not directly linked to our database, and therefore, any explanation for the engagement disparities based solely on academic rank would be speculative. We do acknowledge and elaborate on this point in our Results and Discussion sections. Still, we believe that such nuances may not be suitable for the Abstract, where brevity and clarity are essential. Furthermore, in addition to the slightly longer response time, the conclusion regarding decreased engagement among women REs is also rooted in the observation of a slightly lower response rate compared to men REs. This is a factual description of our findings and by no means it assigns blame or implies any fault on the part of women. Rather, we believe it reflects a system that does not adequately account for women's characteristics and needs, as we thoroughly discuss in the later sections of the paper.

We trust that these clarifications provide a better understanding of our approach and the considerations behind our Abstract's content.

2. Abstract Line 11—SE that are men assign to men—add “(77%)”, “and likewise for women (41%).” To show that although both men and women tend to choose RE of the same sex, that phenomenon is much more prevalent in men.

**Reply**:

We have now incorporated this information into the Abstract, along with the assignment rates without gender specification for context. It is important to include these reference gender base rates to avoid statistical misrepresentation and provide an accurate reflection of the data.

*We find evidence of gender homophily when Senior Editors assign full submissions to Reviewing Editors (i.e., men are more likely to assign full submissions to other men (77% compared to the base assignment rate to men RE of 70%), and likewise for women (41% compared to women RE base assignment rate of 30%)).*

3. Abstract Line 15—remove all following the word “suggest”. Add “Evidence from this intense eLife editorial gender-related review suggests that increasing the proportion of Senior Editors who are women, delaying discussions until all RE are engaged, and making all aware of the potential gender bias in men’s homophily will increase gender equity and enhance academic publication.”

**Reply**:

We thank the Reviewer for this suggestion, which we have incorporated into the abstract, with a slightly different wording.

*Together, our findings confirm that gender disparities exist along the editorial process and suggest that merely increasing the proportion of women might not be sufficient to eliminate this bias. Instead, measures accounting for women's circumstances and needs (e.g., delaying discussions until all RE are engaged) and raising editorial awareness may be needed to increase gender equity and advance academic publication.*

4. Lines 104-6 This prediction has no referencing and sounds like a biased pre-conception. Please add evidence for this or remove it.

**Reply**:

This prediction is based on the entire literature reviewed in the Introduction, which shows a consistent disparity in the participation of women in different roles and processes of scientific publishing [(e.g., Dworkin et al., 2020; Helmer et al., 2017; Murray et al., 2019; Palser et al., 2022)](https://paperpile.com/c/6snec5/ccQk+pcnK+FFLc+aJ20/?prefix=e.g.%2C%20,,,). For example, Helmer and colleagues [(2017)](https://paperpile.com/c/6snec5/FFLc/?noauthor=1) found that women participated less as editors, reviewers and authors in an analysis of the Frontiers series of journals. Moreover, they found substantial homophily in assignment of reviewers by editors and showed that homophily will persist even if numerical parity between genders is reached. Based on this evidence we predicted that this trend will also be found in eLife when examining gender balance in between-Editors interactions and in the engagement in the editorial process.

We followed the reviewer’s suggestion and added those references to the text:

*Based on the literature reviewed above [e.g., 53,54,67,74], we predicted that despite efforts to increase the involvement of women in the BRE, women’s editorial activities would be lower in comparison to men, even after taking into consideration their proportional disparity in the editorial system.*

**References:**

[Dworkin, J. D., Linn, K. A., Teich, E. G., Zurn, P., Shinohara, R. T., & Bassett, D. S. (2020). The extent and drivers of gender imbalance in neuroscience reference lists. Nature Neuroscience, 23(8), 918–926.](http://paperpile.com/b/6snec5/ccQk)

[Helmer, M., Schottdorf, M., Neef, A., & Battaglia, D. (2017). Gender bias in scholarly peer review. eLife, 6. doi:](http://paperpile.com/b/6snec5/FFLc) [10.7554/eLife.21718](http://dx.doi.org/10.7554/eLife.21718)

[Murray, D., Siler, K., Larivière, V., Chan, W. M., Collings, A. M., Raymond, J., & Sugimoto, C. R. (2019). Author-Reviewer Homophily in Peer Review.](http://paperpile.com/b/6snec5/pcnK)

[Palser, E. R., Lazerwitz, M., & Fotopoulou, A. (2022). Gender and geographical disparity in editorial boards of journals in psychology and neuroscience. Nature Neuroscience, 25(3), 272–279. doi:](http://paperpile.com/b/6snec5/aJ20) [10.1038/s41593-022-01012-w](http://dx.doi.org/10.1038/s41593-022-01012-w)

5. Relating to data shown on lines 200-206—there needs to be a section on Limitations in the Discussion. Add this: “Although these RE response rate differences between women and men were statistically significant, this is likely because of the large numbers in this analysis. The actual response differences between 88% of women versus 91% of men are very small. Likewise, the longer response time, again significant due to large numbers is, in reality only a delay of 7 hours for women. This is a minimal delay and unlikely in usual review circumstances to be of practical importance.”

**Reply**:

Regretfully, we cannot comply with this request, as it departs from statistical guidelines and is grounded in a speculative assumption. The core issue lies in the assumption that our findings are significant solely due to the use of a large sample. Our study strictly adheres to rigorous statistical analysis practices, and our conclusions have been derived through meticulous data examination. Dismissing the significance of our findings by attributing them solely to sample size is not aligned with established statistical guidelines.

Furthermore, our analysis encompasses the estimation of effect sizes (Hedges's g) and a comprehensive exploration of the implications of these small effects. Although these effects may be relatively small (Hedges's g = 0.20 and Hedges's g = -0.22), they hold substantial implications within the context of eLife's specific reviewing process, characterised by an online real-time discussion platform.

It is of utmost importance to recognize that our concern extends beyond this methodological aspect. Attributing the significance of our findings solely to sample size, as suggested, could potentially undermine the nuanced and valuable insights provided by our research. Such an attribution might lead to an unfounded conclusion that there is no need to improve gender balance in the editorial process, consequently jeopardising the acceptance of our proposed corrective measures. These measures, which are directly informed by these findings, have also received endorsement within the Reviewer's comments, underscoring their importance and relevance.

6. Lines 210-213 re: submissions/month handled by women (.40) and men (.44). In a section on limitations in the Discussion add this: “Although women than men RE handled fewer submissions per month, common sense says that the difference between .40 and .44 manuscripts/month is unlikely to be important. This difference only reached statistical significance because of large numbers in the analysis.”

**Reply**:

Please see our previous reply to comment #5.

7. Referring to lines 216-222—SE referrals to RE by gender. Add to a limitations section in the Discussion: “However, the 41% that women SE assign to women RE is far less than the 77% that men SE assign to men RE. Thus, homophily is not entirely but largely a factor of men choosing other men.

**Reply**:

We understand the Reviewer’s concern, but we respectfully maintain our position on this matter. As previously discussed in an earlier review round, it is crucial to contextualise the observed assignment patterns in the broader context of gender representation among Reviewing Editors (REs). Assignments made by Senior Editors (SEs) to REs should be evaluated relative to the availability of women REs in the pool. To illustrate this point, let us consider a hypothetical scenario in which there are no men REs available. In such a situation, if women SEs assign 100% of manuscripts to women REs, it would not necessarily reflect gender-related homophily but rather be a consequence of the 100% base rate of women REs. In this specific analysis, we aim to evaluate the extent of homophily beyond the effect of underrepresentation of women REs. Thus, we must consider the base assignment rate. The 41% assignment rate of women SEs to women REs should be compared to the base assignment rate of 30% to women REs, and the 77% assignment rate of men SEs to men REs should be compared to the base assignment rate of 70% to men REs. This comparison reveals an intriguing pattern: women SEs deviate more significantly from the base rate in their homophilic assignments (14%) than men SEs do (10%). This noteworthy difference is consistently reflected across different disciplines, as demonstrated in our contingency table analyses. We hope this clarification helps elucidate our perspective on this matter. We are committed to addressing any remaining concerns or questions you may have.

8. Section describing the eLife database (Lines 310-338) needs to be added to the Introduction. It is awkward and not appropriate as part of the discussion.

**Reply**:

While we value the Reviewer’s input, we respectfully disagree with this particular recommendation. The section in question was intentionally placed in the Discussion to address the limitations of our datasets, which is a conventional practice in scientific writing. Additionally, we would like to clarify that the full details of the eLife database, including its limitations, are already included in the Methods section. Placing this section in the Introduction may disrupt the flow and structure of the paper. By keeping the description of the eLife database in the Discussion, we ensure that readers can connect the specific details of our research to the broader context of the eLife database and understand the implications of the limitations more effectively.

9. Add to Table 3: Lines 350-351—the suggestion here (“To distribute the influence more fairly, a potential solution is to cap the number of consultations per individual RE.”)

**Reply**:

Done with thanks.

10. Remove Lines 382-384—this is re-iterating a bias that is so minimal as to be unimportant.

**Reply**:

As we discussed in response to Comment #5, we acknowledge that the effects described in these lines may appear relatively small when viewed in isolation. However, it is essential to contextualise these effects within the specific framework of eLife's reviewing process, which is characterised by an online real-time discussion platform. In this unique setting, even seemingly small biases can have substantial implications for the overall dynamics and outcomes of the review process. Given this context, we believe it is important to retain the discussion of these effects to provide a comprehensive understanding of the challenges and dynamics at play within the eLife editorial process. We hope this explanation clarifies our position on the matter.

11. Lines 398-399—this suggestion needs to be added to Table 3.

**Reply**:

We appreciate the Reviewer's suggestion regarding the addition of the recommendation to provide gender-specific statistics to Senior Editors about disproportional engagement by gender (Lines 398-399). We would like to clarify that this suggestion has already been incorporated into Table 3 (now Table 2) under the entry: "provide feedback on gender imbalance patterns for Senior Editors."

In our revised table, we have expanded upon this point to enhance clarity, and it now reads as follows: *provide feedback on gender imbalance patterns for Senior Editors (e.g., statistics about disproportionate RE engagement by gender).*

12. Line 444—add this suggestion to Table 3.

**Reply**:

We thank the Reviewer for pointing this out. We added this point to the revised Table 3:

*Increase the proportion of women Senior Editors*

13. Line 463-464—add this suggested improvement to Table 3.

**Reply**:

We thank the Reviewer for pointing this out. We added this point to the revised Table 3 (now Table 2):

*diversify the network of the Senior Editors within the BRE*

14. Revisions to Table 3 (as in #s 11, 12, 13) plus, separate the women with add “slightly” longer response times and the “slightly fewer women responders” into two discrete sections.

**Reply**:

We thank the Reviewer for spotting this omission. We corrected this point in the revised Table 3 (now Table 2):

*Women REs take slightly longer to respond to initial consultations;*

*Women REs respond slightly less frequently to initial consultations*

15. Revisions to Table 3 Change the wording in the remediation section on homophily to: “Increase transparency and awareness to the risks of men’s homophily on the potential gender bias in science.”

**Reply**:

We respectfully regret that we cannot adopt this specific recommendation, as it may not fully align with the empirical findings presented in our study. As we discussed in response to Comment #7, our study reveals that homophily exists among both men and women Senior Editors, although it may stem from different drivers, as we elaborate on in the Discussion section. However, we want to emphasise unequivocally that we firmly assert that all forms of homophily, regardless of the gender of the individuals involved, represent a potential risk to the scientific community. Homophily can restrict diversity, hinder inclusivity, and diminish critical thinking—all of which are indispensable for the advancement of science. Therefore, we maintain that the existing wording appropriately conveys the overarching risk associated with homophily in science, encompassing both men's and women's homophily.

16. Table 3—change the last line of the description under the table to: “These proposed solutions aim to provide potential guidance to stakeholders, enabling them to adopt a proactive and practical approach towards enhancing gender equity in editorial activities.”

**Reply**:

Done with thanks.

17. Line 213—start a new paragraph with the word, “Next”

**Reply**:

We appreciate the suggestion to start a new paragraph with the word "Next" at Line 213. However, we would like to point out that the word "Next" already appears in the preceding line (Line 212), which serves as the introduction to the content presented in Line 213 (*We next explored the effect of the Senior Editor's gender on manuscript assignment to women and men REs*). Given this existing transition, we believe that beginning a new paragraph with "Next" in Line 213 may not be necessary and could potentially disrupt the flow of the text.

18. Line 96—“data” is a plural word—please correct

**Reply**:

We thank the Reviewer for catching the typo at Line 96, where "data" should indeed be treated as a plural word. We would like to reassure them that we are aware of this grammatical rule, as evidenced by multiple instances throughout the text where "data" is correctly used in the plural form, such as "data were pooled" (Line 154) and "These data" (Line 182).

19. Wherever in the document the journal, eLife, is mentioned, it should be in italics; this is standard for publications.

**Reply**:

Done with thanks.

20. Line 467—“manuscript” should be “manuscripts”

**Reply**:

Corrected with thanks (line 483).
