# Supplementary material for: Gender imbalances in the editorial activities of a selective journal run by academic editors
Source: PLoS One. 2023 Dec 11;18(12):e0294805. doi: 10.1371/journal.pone.0294805 (PMC10712860; doi:10.1371/journal.pone.0294805)
Supplement: Attachment — Submitted filename: Rebuttal letter rev4.docx [file pone.0294805.s007.docx]

Rebuttal letter

Reviewer #2: This manuscript still needs a limitation section in the discussion, as is standard in all research publications of which I am aware.

Please add:
"It is a limitation that the level of academic achievement for all of the women and men in the data were not included at the same time as the primary data analysis. This has left an implied gender equity academic status that is likely not correct. The sociology of gender-related behaviour predicts that academic stature as well as gender likely altered the outcome of meetings in which manuscripts were evaluated."

Reply: We thank the reviewer for this comment, which we have added to the revised Discussed that now reads as follows:

*Unfortunately, the fact that our primary datasets lack direct information on academic attainment levels for all women and men is a limitation. This lack of information should not be interpreted to mean that academic status is equal across genders in our datasets, an assumption that is likely to be incorrect. The sociology of gendered behavior predicts that both academic status and gender likely influenced the outcome of the interaction in which manuscripts were evaluated, as discussed below.*
